# Injuries to the Epiphyses and Their Results

**Published:** 1893-11

**Authors:** 


INJURIES TO THE EPIPHYSES AND THEIR RESULTS.—In an interesting lecture delivered at the Royal College of Surgeons, Jonathan Hutchinson, Jr. (*British Medical Journal*, 1893, No. 1697), calls attention to the fact that injuries to the epiphyses is more frequent than is usually taught. He bases his observations on 350 cases, nearly 150 of which have not been published before. These injuries are of especial importance, because of the difficulty in diagnosis and the danger of arrest of development in the injured bone. The latter result is most prejudical when one of the bones of the forearm or leg is affected. The author has shown by experiments on rabbits that the epiphysial disk has a remarkable vitality, and that when the epiphysis is accurately replaced, after being separated, the natural development of the limb usually follows. It is important to effect reduction as early as possible, because: 1. The rapid occurrence of swelling will make the diagnosis and correction of the exact displacement more difficult. 2. The connecting bridge of periosteum quickly thickens and shortens; from this cause, at the post-mortem of a rabbit, whose lower femoral epiphysis had been detached and allowed to remain displaced, it was found quite impossible by any reasonable force to effect reduction, although only eight days had elapsed since the injury. 3. The more prompt the replacement the less will be the risk of exuberant callus, of interference of growth, and possibly also of suppuration and necrosis.

The author refers to three "undoubted cases" of separation of the sternal end of the clavicle in patients aged twenty, fourteen, and seventeen years respectively. At about the twenty-fifth year the epiphysis becomes fused with the shaft.

In most cases of detachment of the upper epiphysis of the humerus the shoulder-joint is said not to be opened, particularly if the patient is under the age of twenty. The author says: "It is sufficienty accurate and a point of practical importance to say that separation of this epiphysis before the age of about twenty takes the place of dislocation of the shoulder-joint. How much trouble in treatment and discredit to the practitioner might have been saved if this fact was generally admitted. Time after time the doctor called to a case of separation of this epiphysis has pronounced it to be one of dislocation, has employed various methods of reduction, and has been mortified by the tendency to recurrence and the very indifferent result obtaned."

In analyzing the records of 26 cases of this injury, 13 of which were observed personally, in 6 instances the accident was found to have occurred at birth, and in 4 during the first year of life. Omitting these the average age at which the detachment occurred was thirteen years; in 17 cases the patient was fifteen years of age or above. The following are given as the chief points in the diagnosis of the separation of this epiphysis:

1. The age of the patient—under about twenty years.

2. The arm is comparatively helpless, the elbow often directed a little outward or backward.

3· Abnormal mobility just below the shoulder-joint, best made out by abducting the humerus.

4. Rapid swelling about the shoulder, some shortening if the diaphysis is wholly displaced.

5. Muffled crepitus on replacement.

Reduction is best accomplished by steady traction on the arm, slight abduction, aided by rotary movement, or by direct pressure. If the diaphysis protrudes just under the skin, and prolonged efforts at reduction fail, the end of the diaphysis should be exposed (with every precaution to asepsis), the opening in the periosteal sheath enlarged, or the rent in the other soft tissues held open, and the bone returned to its place. This has been done with success, even at the interval of several weeks from the accident; but it is then usually necessary to resect part of the diaphysis.—*Amer. Jour. Med. Science.*

